# The dendritic engram

**DOI:** 10.3389/fnbeh.2023.1212139

**Published:** 2023-07-27

**Authors:** George Kastellakis, Simone Tasciotti, Ioanna Pandi, Panayiota Poirazi

**Affiliations:** ^1^Institute of Molecular Biology and Biotechnology, Foundation for Research and Technology, Heraklion, Greece; ^2^Department of Biology, University of Crete, Heraklion, Greece

**Keywords:** memory, dendrites, engram, plasticity, modeling

## Abstract

Accumulating evidence from a wide range of studies, including behavioral, cellular, molecular and computational findings, support a key role of dendrites in the encoding and recall of new memories. Dendrites can integrate synaptic inputs in non-linear ways, provide the substrate for local protein synthesis and facilitate the orchestration of signaling pathways that regulate local synaptic plasticity. These capabilities allow them to act as a second layer of computation within the neuron and serve as the fundamental unit of plasticity. As such, dendrites are integral parts of the memory engram, namely the physical representation of memories in the brain and are increasingly studied during learning tasks. Here, we review experimental and computational studies that support a novel, dendritic view of the memory engram that is centered on non-linear dendritic branches as elementary memory units. We highlight the potential implications of dendritic engrams for the learning and memory field and discuss future research directions.

## 1. Introduction

Early theories of memory did not take into account the computational properties afforded by dendrites. The classical connectionist model of memory engrams relies on Hebbian plasticity through LTP and LTD which results in the strengthening and weakening of synapses in the neuronal assembly that is believed to encode a memory. Changes in synaptic connectivity are impacted, however, by their hosting structure, which is the dendritic branch. Computational studies which modeled dendritic trees (Rall, 1962) pioneered the study of signal propagation in dendrites and their responses. Dendrites are capable of non-linear integration of inputs and generate all-or-none electrical excitation known as dendritic spikes. Computational models have been used to explore the conditions for their initiation and their propagation (Segev and Rall, 1998). Synapse clustering in small areas of dendrites in combination with NMDA receptor activation was shown to confer non-linear problem solving capabilities to dendrites (Mel, 1992). Further work showed that the spatiotemporal arrangement of synaptic inputs on dendrites plays a crucial role, as it affects both the computational and the storage capacity of neurons (Poirazi and Mel, 2001; Papoutsi et al., 2014; Kastellakis et al., 2015). On this basis, it was proposed that dendrites can be modeled as a second layer of computation within neurons (Poirazi et al., 2003b). Since then, experimental studies have validated many of those predictions, including observations of clustering of synaptic contacts into functional (Holtmaat and Svoboda, 2009; Kleindienst et al., 2011; Takahashi et al., 2012; Ishikawa and Ikegaya, 2020; Adoff et al., 2021; Hwang et al., 2022) and anatomical groups (Frank et al., 2018; Harris, 2020), and the role of dendritic spikes and dendritic depolarization in the induction of plasticity (Polsky et al., 2004; Spruston, 2008; Gómez González et al., 2011; Lavzin et al., 2012). The culmination of experimental and theoretical studies on the role of dendrites in brain functions resulted in the proposition that dendritic branches serve as the fundamental functional units in the brain (Govindarajan et al., 2006; Branco and Häusser, 2010).

Given the experimental and computational evidence that dendrites are key contributors to many memory-related processes, it becomes evident that memory engrams must also be characterized at the dendritic level. The main mechanism behind memory engram formation is synaptic plasticity (Josselyn et al., 2015; Choi et al., 2018). Synaptic plasticity alters the strength of individual synapses in response to learning and shapes what is called the “synaptic engram” of a given memory. However, as synapses are located within dendrites, physical changes in other dendritic mechanisms (e.g., ionic conductances in dendritic shafts) cannot be separated from those taking place in synapses. This is simply because memory-induced synaptic changes would be very different if synapses did not impinge on dendrites. For example, dendritic ionic mechanisms that drive localized spikes result in stronger LTP at a given synapse, compared to what the same synapse would undergo if those dendritic spikes did not occur (Golding et al., 2002). Moreover, due to the anatomical compartmentalization of dendritic branches, synaptic potentiation/depression can be spatially restricted to specific compartments while neighboring synapses can benefit from cooperative plasticity effects (Spruston, 2008; Makara et al., 2009; Govindarajan et al., 2010). Finally, the intrinsic excitability of dendritic sub-trees can also undergo plastic changes (e.g., the conductance of A-type K+ channels) which affect their ability to drive local spikes, somatic firing and bursting (Losonczy et al., 2008). Given the above, we propose that the compartmentalization into non-linear dendritic units in which modifiable synapses reside comprises the *dendritic engram*. More specifically, instead of considering synapses as the memory unit as typically assumed in connectionist models, we suggest that *compartmentalized non-linear dendritic branches* serve as the memory unit.

Understanding dendritic engrams is important as they are directly linked to the computational power of neuronal circuits. For instance, if we were to count all different synaptic strength configurations (presumably produced by learning) in a single neuron –while ignoring the impact of dendritic non-linearities- it would only account for a small number of all possible memories that can be stored within this neuron (Poirazi and Mel, 2001). This is because a given set of synaptic inputs can induce a wide range of different neuronal outputs, depending on the ionic and anatomical characteristics of the dendrites in which they reside and their proximity to other synapses (Poirazi and Mel, 2001; Poirazi et al., 2003a). Thus, synaptic configurations alone comprise of only a small part of all potential memory engrams.

This article explores the plausibility and the consequences of the dendritic engram hypothesis. Toward this goal, we review evidence that support the key role of dendrites in memory processes, the molecular mechanisms underlying these processes and the computational advantages that dendrites provide.

## 2. Experimental evidence for the role of dendrites in engrams

**FIGURE 1:**
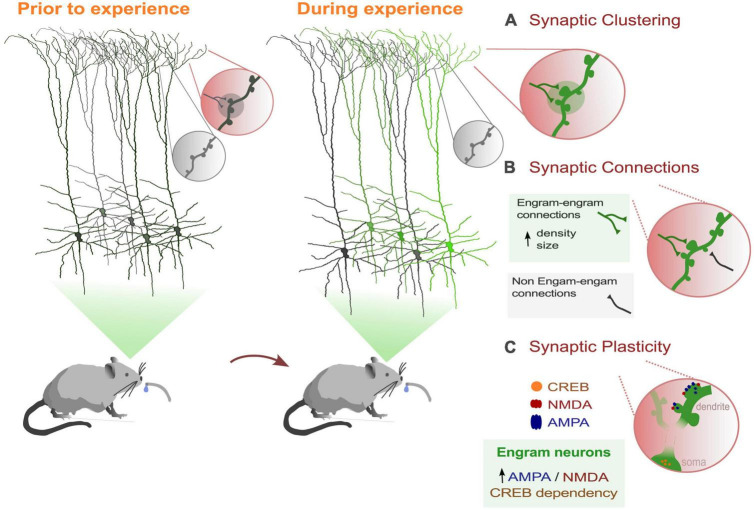
Dendritic dynamics that support memory processes in behaviorally-related engram cells. **(A)** Learning induces the selective formations and strengthening of a subset of synaptic connections in a clustered form. **(B)** Engram dendrites showed greater synaptic strength and increased spine density than non-engram cells. **(C)** Engram cells show greater AMPA/NMDA current ratios than non-engram cells. Also, activation of the transcription factor CREB (cAMP-responsive element binding protein) facilitates memory processes and increases spine density (Sargin et al., 2013).

In recent decades, memory research has undergone a revolution with the use of new genetic tools as endogenous markers of neuronal activity. Immediate-early gene (IEG) promoters have enabled researchers to target and manipulate neurons thought to encode a specific behavior, also known as engram neurons, during a wide range of tasks, and within a specific time window induced by the experimenter (Cruz et al., 2013; Josselyn et al., 2015). The ability to manipulate memories has allowed numerous engram studies to address long-standing questions about the cellular and network dynamics that facilitate the formation and reactivation of engram networks. Despite the important role that dendrites play in processing information received from the majority of synaptic inputs to the neuron, only a limited number of studies have focused on dendrites as the centerpiece of the engram. In this section, we aim to emphasize the properties of dendrites and the changes in dendritic dynamics associated with behavior, including plasticity. These converging lines of evidence suggest that a dendritic branch could function as a unit of memory (Figure 1).

### 2.1. Active dendritic computations during behavior

In order to survive, animals must gather and process a variety of information from their environment. The integration of these information streams can be selectively achieved through cellular and dendritic mechanisms. Dendrites possess unique electrical characteristics that allow neurons to generate spikes by processing synaptic inputs in diverse ways. Dendritic NMDA and calcium spikes/plateaus can induce somatic action potentials (Larkum et al., 1999) or lead to somatic burst firing (Polsky et al., 2004). Additionally, dendritic spikes elevate the concentration of calcium within the dendritic branch and subsequently within the cell, triggering synaptic plasticity (Flavell and Greenberg, 2008). Through plasticity, dendritic synapses are formed in selective and clustered ways to facilitate the processing and storage of information. As such, dendrites utilize a combination of properties to provide the neuron with complex computational abilities.

Experimental studies in rodents that combine behavioral tasks with two-photon Ca2+ imaging of pyramidal dendrites have provided valuable insights into the contribution of dendrites to complex behaviors. The findings from these studies demonstrate that apical dendritic activity represents features that are relevant to an animal’s behavior (Xu et al., 2012; Sheffield and Dombeck, 2015; Takahashi et al., 2016; Ranganathan et al., 2018; Kerlin et al., 2019; O’Hare et al., 2022). While most studies using calcium imaging have found that somatic and dendritic activity are highly correlated during behavior (Ranganathan et al., 2018; Beaulieu-Laroche et al., 2019), there is also evidence that dendrites support independent operations (Francioni et al., 2019; Kerlin et al., 2019; Voigts and Harnett, 2020). Notably, different motor learning tasks (e.g., forward/backward running on a rotarod) have been shown to cause calcium transients in different, non-overlapping dendritic branches (Cichon and Gan, 2015), and task-associated calcium signals in dendrites are compartmentalized and clustered within dendritic branches (Kerlin et al., 2019). These experimental approaches suggest that dendritic branches are capable of independent local operations (Poirazi et al., 2003b; Polsky et al., 2004) and may serve as distinct engram units.

### 2.2. Behavior-related dendritic structural plasticity

Pyramidal neurons are the predominant excitatory cells in the brain, characterized by their complex dendritic arbors and small protrusions known as dendritic spines. These spines host the majority of the excitatory synapses in the brain, and their density and size play a critical role in determining the synaptic input that a neuron can receive. Importantly, dendritic spines are capable of undergoing structural changes in response to new experiences, and these changes are essential for the processes of learning and memory (Holtmaat et al., 2005; Zuo et al., 2005).

Dendritic spine dynamics can be classified into two main categories: temporal dynamics and spatial dynamics. Temporal dynamics refer to how the turnover of spines changes over time and in response to behavior. Spatial dynamics, on the other hand, describe the relative influence of existing spines on spine dynamics, such as their role in promoting the formation, elimination, or clustering of new spines (Holtmaat et al., 2005).

A growing body of research suggests that changes in the size and density of dendritic spines are closely linked to the formation of memories (Lamprecht and LeDoux, 2004). One well-studied example is fear learning, where studies have shown that fear conditioning leads to a long-lasting increase in spine elimination in the frontal association cortex. Conversely, fear extinction triggers the formation of newly formed spines, which interestingly tend to appear near previously eliminated spines (Lai et al., 2012).

In contrast, neurons in the auditory cortex, which are involved in fear memory recall, respond to auditory fear conditioning by increasing spine formation. Notably, recent research has shown that newly formed spines induced by fear conditioning with one auditory cue tend to cluster within dendritic branch segments and are spatially segregated from new spines induced by fear conditioning with a different auditory cue (Lai et al., 2018). These findings suggest that learning-induced spine dynamics occur at the level of specific spines and dendritic branches in various brain regions, including the association (Lai et al., 2012), auditory (Lai et al., 2018), visual (El-Boustani et al., 2018), and motor cortex (Xu et al., 2009).

Going further, several studies report that behavior-related alteration of spine dynamics occur, not only in non-random locations, but also in spatial proximity to other potentiated spines, forming clusters of spines within dendrites. We refer to them as “dendritic hotspots” (Kastellakis et al., 2015). *In vivo* studies have shown that learning induces the formation of new spines in a clustered manner (Fu et al., 2012; Frank et al., 2018). Such clustering is important for local dendritic computations and can facilitate the generation of dendritic plateau potentials increasing the efficiency of information storage (Poirazi et al., 2003a; Kastellakis et al., 2015; Frank et al., 2018). In a simple learning protocol, if two memories are linked within the same dendritic branch, the potentiation of one memory may result in a corresponding potentiation of the other memory. Synaptic plasticity mechanisms operating locally within the dendritic branch can link memories encoded close in time, directing storage into overlapping ensembles, as reported by experimental (Cai et al., 2016) and computational studies (Kastellakis et al., 2016). Overall, the selective strengthening of a subset of synaptic connections (clusters) within the same dendritic branch, rather than randomly allocated synapses across different dendritic branches, may serve as an efficient mechanism for the activation of dendritic branches as an engram unit.

### 2.3. Dendritic dynamics in labeled engram cells

Recent studies are utilizing learning-dependent cell labeling, known as *engram labeling* to examine the role of dendrites in memory formation, specifically within behavior-related labeled engram dendrites. For instance, (Ryan et al., 2015) demonstrated that 1 day after fear conditioning, labeled dentate gyrus engram dendrites exhibited greater synaptic strength and increased spine density compared to non-engram DG cells. In another recent study, the synaptic connections between CA3 and CA1 engram cells were labeled using a novel technique called “dual-eGRASP.” The results showed that the CA1 engram cells which receive inputs from CA3 engram cells had a significantly higher number and larger size of spines compared to non-engram synapses. This increased connectivity was also found to be associated with improved memory strength (Choi et al., 2018).

Further supporting the importance of dendrites in memory engrams, (Hwang et al., 2022) conducted a recent study revealing that dendritic spine density increases and newly formed spines stabilize exclusively on engram primary motor cortex (M1) neurons, with no similar changes observed in neighboring non-tagged neurons. Additionally, motor learning results in increased strength of output from M1 engram neurons to the postsynaptic striatal neurons by forming local clusters along the dendrites (Hwang et al., 2022; Figure 1A). These findings further suggest that dendritic hotspots play a crucial role in memory engrams by enabling the selective strengthening of a subset of synaptic connections within engram dendritic units.

Finally, few studies have investigated the sub-cellular mechanisms of localized forms of plasticity in dendrites that facilitate memory formation of labeled engrams. As we mentioned above, overlapping dendritic segments were activated when encoding memories that are experienced close in time. Dendritic co-allocation of memories was found in dendritic segments, such that memories linked in time are likely to be allocated to the same dendritic segments (Sehgal et al., 2021). Using a model, the authors demonstrated that dendritic mechanisms such as dendritic excitability and plasticity are necessary for linking memories acquired close in time. In addition, a recent study showed that synaptic plasticity between engram connections is likely involved in the formation of remote memories. In particular, suppressing the activity of CREB in mPFC (medial Prefrontal Cortex) engram neurons disrupts the recall of these memories (Matos et al., 2019; Figure 1C). Enhancing the function of CREB has been shown to boost the density of dendritic spines (Marie et al., 2005), which could be a potential plasticity mechanism underlying engram formation. Finally, (Ryan et al., 2015) found that 1 day after fear conditioning, labeled dentate gyrus engram dendrites not only exhibited greater synaptic strength, but also a higher AMPA to NMDA ratio, highlighting the importance of dendritic plasticity mechanisms underlying engram memory formation.

The aforementioned studies suggest that dendritic plasticity in engram neurons is implicated in the process of forming memories. Thus, it is important to understand how and which dendritic plasticity mechanisms may underlie the formation of memory engrams.

## 3. Molecular and plasticity mechanisms in dendrites

**FIGURE 2:**
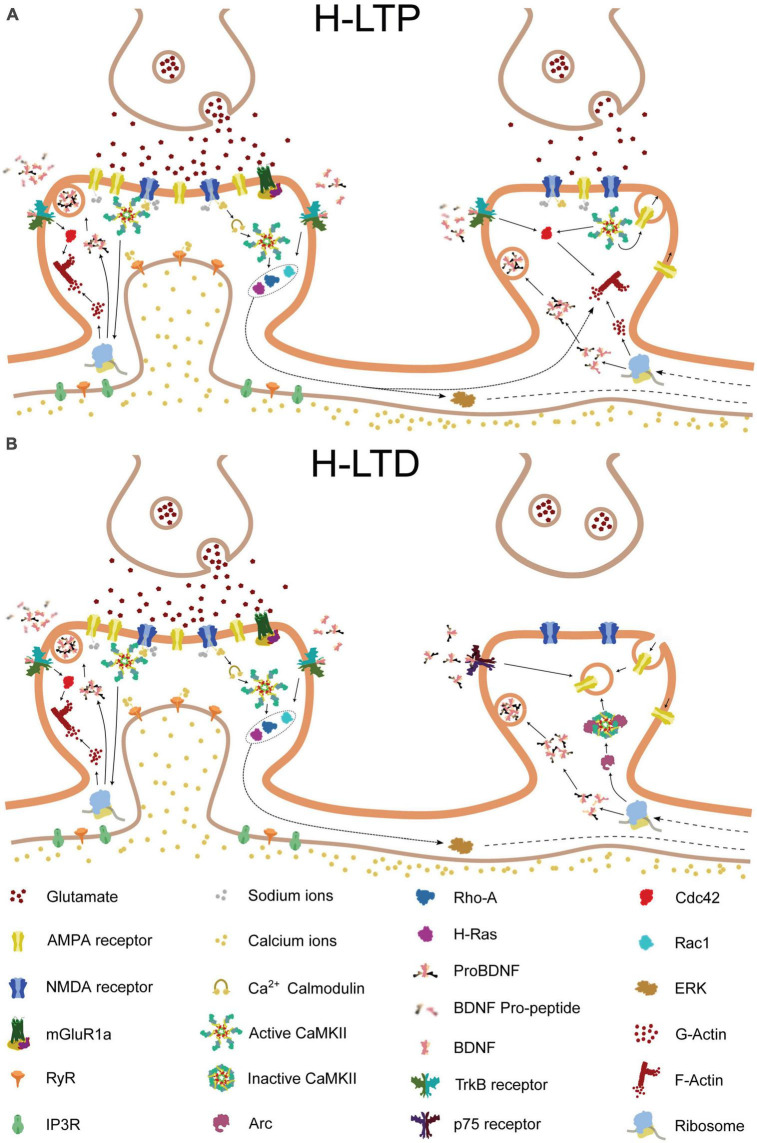
Molecular pathways of heterosynaptic potentiation and depression. The molecular pathways involved in heterosynaptic potentiation and depression, driven by homosynaptic potentiation at a neighboring spine. Glutamate release from the presynaptic terminal activates AMPA and NMDA receptors on the postsynaptic membrane of the central spine, resulting in calcium influx into the synapse. The calcium influx, in combination with calmodulin, leads to the activation of Ca2 + -calmodulin-dependent protein kinase II (CaMKII). Activated CaMKII, in turn, activates Ras and RhoA1, and, through brain-derived neurotrophic factor (BDNF) and its receptor TrkB, also activates Cdc42 and Rac1. Additionally, CaMKII and BDNF activation can lead to the local translation of Arc mRNA that was previously present. Activated Cdc42 remains confined to the central spine, whereas Ras, RhoA1, Rac1, and Arc can spread along the dendritic shaft and potentially interact with neighboring spines. **(A)** If a nearby spine is inactive, Arc is recruited to the spine through an interaction with inactive CaMKIIβ. Alternatively, release of proBDNF by the activated central spine, in the absence of tissue plasminogen activator (tPA)/plasminogen system, can result in binding of proBDNF to p75 neurotrophin receptors (p75NTR). These processes can promote either structural spine shrinkage or endocytosis of surface AMPA receptors, leading to heterosynaptic long-term depression (H-LTD). **(B)** In contrast, if a neighboring synapse is instead activated, tPA promotes the cleavage of proBDNF to BDNF, which binds to TrkB receptors. This, in combination with NMDA receptor-driven CaMKII activation, leads to Cdc42 activation. Cdc42 activation, in turn, together with the spread of activated Ras, RhoA1, and Rac1 from the neighboring synapse, drives the remodeling of the actin cytoskeleton, leading to structural heterosynaptic long-term potentiation (H-LTP).

One theory for how engram networks persist and reactivate during learning is by strengthening specific synaptic connections between neurons that fire in synchrony, as suggested by Hebb (1949). We speculate, however, that the selective strengthening of a subset of synaptic connections and the emergence of branch specificity may be facilitated by synaptic plasticity mechanisms such as heterosynaptic plasticity. These mechanisms could provide dendritic braches with the ability to leverage their complex properties during repeated learning, thereby functioning as a unit of memory engram (Figure 2).

### 3.1. Dendritic compartmentalization

Dendritic compartmentalization entails that individual dendritic branches of a neuron can operate as computational subunits, each with its own input and output function. One of the key mechanisms underlying dendritic compartmentalization is dendritic spike generation. This refers to the ability of dendrites to generate action potentials mediated by various conductances, including N-methyl-D-aspartate receptors (NMDAR) (Schiller et al., 2000; Larkum et al., 2009), voltage-gated sodium (Herreras, 1990; Kim and Connors, 1993; Schiller et al., 1997; Kamondi et al., 1998) or calcium channels (Larkum et al., 1999), and which allow for local processing of information within the dendrite. Dendritic compartmentalization can also be facilitated by branch-specific inhibition. For example, shunting inhibition near a branching point can hinder the propagation of excitatory inputs to nearby branches or even prevent the initiation of a dendritic spike (Mel and Schiller, 2004; Gidon and Segev, 2012; Wilmes et al., 2016).

Dendritic compartmentalization in individual neurons can have a profound effect on network computations, by allowing for more complex and sophisticated processing of information. For example, it can modulate activity synchronization by regulating the spread of dendritic spikes across the network (Spratling and Johnson, 2001). Dendritic compartmentalization has been implicated in cognitive processes such as learning and memory. Studies have indeed shown that the occurrence of dendritic spikes generated in response to specific inputs can be important for the induction of plasticity (Golding et al., 2002; Holthoff et al., 2004; Losonczy and Magee, 2006; Nevian et al., 2007) which is the basis of engram formation, and it has been demonstrated that the dendritic branch could be thought as the functional unit of synaptic integration and plasticity (Harvey and Svoboda, 2007; Branco and Häusser, 2010).

### 3.2. Synaptic cooperativity in dendrites

While the most studied form of plasticity is homosynaptic (Hebbian) plasticity as already mentioned, this phenomenon cannot be considered in isolation from its surrounding environment. The non-linear events that occur in dendritic compartments ensure that the impact of a specific input pattern cannot be disentangled from the inputs arriving at neighboring synapses. Additionally, the lateral spread of some messenger molecules within the dendritic branch (Engert and Bonhoeffer, 1997; Harvey and Svoboda, 2007) or the diffusion of pre-synaptically expressed non-specific messengers (Schuman and Madison, 1994; Engert and Bonhoeffer, 1997) contribute to the interdependency of neighboring synapses (usually referred as “cooperativity”). The direct consequence of these phenomena is the breakdown of input specificity for plasticity of synapses that are tens of microns apart. Moreover, early-phase Long Term Potentiation (E-LTP) at one synapse can lower the threshold for E-LTP induction at synapses within a ∼10-μm neighborhood on the same dendritic branch (Harvey and Svoboda, 2007; Yadav et al., 2012). This suggests that the strength of synaptic connections in a dendritic compartment is not only influenced by local, synapse specific events but also by events occurring in surrounding compartments. Corroborating this hypothesis, (Oh et al., 2015) investigated the plasticity of clustered hippocampal CA1 pyramidal synapses using high-frequency glutamate uncaging. The results show that, potentiating simultaneously more than five synapses on neighboring spines causes the weakening and shrinkage of the inactive synapses within the cluster. Moreover, this phenomenon does not emerge from simple resource competition but is a product of a defined molecular pathway. Indeed, they observed that inhibiting different molecules such as calcineurin or Ca2+/calmodulin-dependent protein kinase II (CaMKII), could decouple the structural potentiation from the heterosynaptic shrinkage of the inactive synapses.

It is evident that the non-linear events occurring in dendritic branches ensure high compartmentalization of information processing, while nearby synapses can influence each other through a variety of electrical and molecular interactions. To show that dendritic compartmentalization and synaptic cooperativity play a crucial role in the storage of information under physiological conditions, (Fu et al., 2012) measured the formation of spine clusters in layer 5 (L5) motor cortex pyramidal neurons of mice during motor training. Interestingly, not only there was a strong bias for new spines to form in close proximity to a stable spine, but these new clustered spines were more persistent through time compared to their non-clustered counterparts. This was in contrast to the control conditions, in which the bias was to avoid the existing stable spines, and more generally the same dendritic branch. Considering the evidence that neurons store information through synaptic clustering, it is necessary to explore the molecular cascades that enable this phenomenon from a mechanistic standpoint.

### 3.3. Localized signaling and positive feedback loop: NMDAR, CaMKII, and BDNF/TrkB in synaptic plasticity

One of the most important effects of synaptic cooperativity is the non-linear NMDAR-mediated amplification of spine calcium signals along individual dendrites, that usually shows a proximodistally increasing gradient (Weber et al., 2016). The magnesium block ensures that NMDA receptors are not activated by low levels of glutamate or weak synaptic inputs. Therefore, it sets a threshold for NMDA receptor activation, requiring a stronger and more synchronous synaptic input to depolarize the membrane potential and relieve the block. Because of this property, the NMDA receptor is widely considered a coincidence detector, a crucial mechanism for regulating synaptic plasticity. During periods of high-frequency synaptic activity, the magnesium block release allows rapid calcium influx, which in turn can trigger intracellular signaling pathways which lead to long-term changes in synaptic strength and plasticity. Cytoplasmatic Ca2+ concentration increase, in fact, is thought to be the starting point for all the chemical cascades that result in input-dependent homo- and heterosynaptic plastic alterations of synapses (Rose et al., 2009; Chu et al., 2015; Hedrick et al., 2016; Lee et al., 2016; Jungenitz et al., 2018; Niculescu et al., 2018; Tazerart et al., 2020).

How does the local increase in the calcium concentration trigger structural changes that involve synapses that are several microns in distance? When Ca2+ enters the synapses, it interacts with Calmodulin to activate the Calcium–calmodulin-dependent protein kinase II (CaMKII). Binding of Ca2+-CaM to the regulatory segment of the protein relieves this inhibition by removing the autoinhibitory segment from the catalytic site, allowing the kinase to become active (Lisman et al., 2012). Interestingly, upon activation of two adjacent subunits, the enzyme starts a process of autophosphorylation on its regulatory domains. This phenomenon leads to the persistent activation of CaMKII that lasts up to several minutes after the Ca2 + -CaM dissociation (Chang et al., 2017). Considering that the Ca2+ transient inside the spine lasts only 0.1 s, the autocatalytic property of this protein is fundamental to initiate the plasticity-related biochemical signal transduction.

CaMKII activity is also strictly correlated with the relative position of the protein in the spine and its interaction with specific CaMK-associated-proteins (CaMKAPs). Several studies using single-particle tracking photoactivated localization microscopy (sptPALM) have shown that CaMKII exhibits highly dynamic behavior in dendritic spines (Lu et al., 2014; Kim et al., 2015). For example, studies have shown that CaMKII molecules move in and out of spines with a half-life of about 2 min, but they also undergo periods of confined diffusion within spines after localized signaling events, indicating that they may interact with specific binding partners (Lee et al., 2009; Lu et al., 2014). More specifically, CaMKII is known to bind NMDAR and this association not only prevents the inactivation of the kinase (prolonging the duration of its active state) but also increases channel conductance, allowing for a greater influx of calcium ions into the neuron. This increased calcium influx can then lead to further activation of CaMKII, creating a positive feedback loop. The close proximity caused by the GluN2B-CaMKII binding to the Post Synaptic Density (PSD) helps this protein in another fundamental function: at this location, CaMKII can phosphorylate nearby AMPA receptors (AMPARs), increasing their conductance and their permanence and translocation rate on the plasma membrane.

Considering the localized nature of the active form of CaMKII and its relatively small activity time window, it is clear that downstream signaling molecules are required to extend the LTP signals and guarantee any form of synaptic cooperation. Several studies showed, through fluorescence resonance energy transfer (FRET), that the Ca2+ influx and the activation of CaMKII in dendritic spines can lead to the localized exocytosis and/or synthesis of Brain-Derived Neurotrophic Factor (BDNF) (Tongiorgi et al., 1997; Brigadski and Leßmann, 2020; Zagrebelsky et al., 2020), a protein that promotes the survival and growth of neurons, as well as synaptic plasticity. BDNF can act on several different types of receptors, but one of the most important is tropomyosin related kinase (TrkB) that is also localized in dendritic spines. Through a series of complex interactions, BDNF is actively transported outside the cell to bind with the external domain of the TrkB receptor localized in the active spine. This event triggers the beginning of an autocrine feedback loop in which BDNF induces the local synthesis and the secretion of more BDNF, facilitating also the transportation and targeting of newly synthesized BDNF mRNA (Harward et al., 2016).

The different pathways that follow TrkB activation are involved in the rise of intracellular Ca2+, mRNA translation, gene expression regulation and facilitation of local protein translation. While studies have shown that ERK signal, a downstream effector of TrkB, is fundamentally involved in the regulation of the cell gene expression (Wiegert et al., 2007; Bramham, 2008), emerging evidence suggests that its involvement may be limited to proximal, short dendrites. Phosphorylated ERK, appears to diffuse to the nucleus, activating transcription factors like CREB to regulate the expression of Immediate Early Genes (IEGs). Its action likely complements other mechanisms, such as calcium oscillations or other biochemical pathways, which are responsible for signaling to the nucleus from synapses in more distal and longer dendrites. However, the exact mechanisms involved remain an open question. Finally, the transcripts of the IEGs are transported back to the dendrites, where they are translated in proximity of spines that have been primed by sufficiently strong inputs. This activity-dependent synthesis of proteins represents a key component underlying synaptic cooperativity.

### 3.4. Small GTPases shape heterosynaptic potentiation

What has been described so far, however, is not enough to explain the heterosynaptic crosstalk in plasticity. One of the fundamental elements allowing synaptic cooperativity in dendrites depends on the activity of a group of small GTPases involved in the regulation of the cytoskeleton dynamics in nearby spines. Even though CaMKII and BDNF-TrkB induce homosynaptic structural changes, they also lead to the activation of these proteins. Specifically, the activation of TrkB by BDNF can trigger a cascade of signaling events that involve Rac1, a member of the Rho family of GTPases. On the other hand, CaMKII activation results in the activation of H-Ras and RhoA1. These proteins are known to promote the formation of new dendritic spines and the enlargement of existing spines, as well as the stabilization of synapses. The activation of these GTPases is usually accompanied by the activation of another downstream effector of BDNF: Cdc42. This protein stays confined to the potentiated spine, promoting local actin remodeling and therefore structural plasticity (Figure 2A).

Using two-photon fluorescence lifetime imaging microscopy (2pFLIM), combined with an optimized FRET-based biosensor, studies have shown that activated H-Ras spreads along the dendrite for ≈ 10 μm and enters neighboring spines instead of being limited to the potentiated spine (Harvey et al., 2008). Moreover, it was found that, while activated H-Ras is required for both homosynaptic potentiation and facilitatory Heterosynaptic-LTP (H-LTP), disrupting H-Ras signaling to ERK only impairs heterosynaptic facilitation. This demonstrates that the functions of H-Ras in homosynaptic and heterosynaptic plasticity can be different depending on its location and the downstream effector it interacts with. With a similar approach, other studies confirmed that also RhoA and Rac1 can diffuse laterally up to 10 μm and invade neighboring spines, although this invasion alone is not sufficient to initiate heterosynaptic plasticity (Murakoshi et al., 2011; Hedrick et al., 2016). Additionally, they showed how the spread of activated Rac1 and RhoA through the dendritic shaft is necessary for H-LTP. Interestingly, these two factors cannot be induced by a subthreshold stimulation, contrarily to Cdc42, which can be activated with a stimulation of mild intensity. Thus, H-LTP requires the diffusion of activated H-Ras, Rac1, and RhoA from the initially potentiated spine, and the activation of Cdc42 in the neighboring spine by a subthreshold stimulus.

To gain a comprehensive understanding of the structural impact of these proteins, it is crucial to also consider their temporal activation profiles. The induction of LTP at a single spine activates all these small GTPases within 1 min. Only the activities of Cdc42 and RhoA are sustained for more than 30 min, while the activity of H-Ras is not sustained (Harvey et al., 2008; Murakoshi et al., 2011). Therefore, the Ca2 + -CaMKII-BDNF/TrkB pathways constitutes a signal transduction system that spans a timescale of milliseconds to more than half an hour causing synapse-specific and heterosynaptic plasticity (Cdc42, Rac1 and RhoA) in dendrites while sending a signal to the cell nucleus (H-Ras).

### 3.5. Tagging inactive synapses: the dendritic mechanisms behind heterosynaptic long-term depression

Activation of small GTPases leads to the activation of actin binding proteins such as Arp2/3 and inactivation of proteins like cofilin (Murakoshi et al., 2011), favoring actin polymerization, branching and stabilization. These cascades can therefore explain, at least partially, the mechanisms underlying the heterosynaptic structural LTP in dendrites. As mentioned before, however, one of the main observations of synaptic cooperativity was the shrinkage of the surrounding, inactive spines (Oh et al., 2015). More generally, the Heterosynaptic Long Term Depression (H-LTD) has been often reported to emerge in several synaptic cooperativity protocols.

To fully understand this phenomenon, it is crucial first to describe a few key elements of the transcriptional regulation that the cell undergoes after the induction of Late-LTP (L-LTP). According to the Synaptic Tag-and-Capture Hypothesis, a sufficiently strong synaptic stimulation activates at least two mechanisms: a protein synthesis independent setting of the local tag and a signal to the nucleus that induces the transcription of IEGs. These newly synthesized Plasticity Related Products (PRPs) are then transported to dendritic spines in an inactive form, are unblocked by the tagged synapses and used (Frey and Morris, 1997; Redondo and Morris, 2011). Even though the H-Ras/ERK pathway has been identified as one of the molecular backbones of the synapto-nuclear signal, the identity of the synaptic tag is still unknown. One of the main hypotheses states that the synaptic tag could be a temporary state of the synapse that involves multiple proteins and their interactions, such as the actin cytoskeleton structure (Redondo and Morris, 2011). One example of this is the stable pool of F-actin that is formed following the induction of long-term potentiation (LTP) (Okamoto et al., 2004; Honkura et al., 2008; Ramachandran and Frey, 2009). Alternatively, other studies propose that TrkB activation, induced by either early or late LTP, may act as a tag to capture PRPs that have been induced by L-LTP, through an independent pathway (Lu et al., 2011). This concept is in line with experimental evidence demonstrating that a weakly stimulated synapse only undergoes structural potentiation when it is in spatiotemporal proximity to a strong stimulation event occurring on another spine.

Importantly, it has been observed that synaptic tagging can occur not only at stimulated spines but also at non-stimulated spines. A study on the immediate early gene (IEG) Arc, which is involved in the endocytosis of AMPA-type glutamate receptors, has proposed the existence of “Inverse Tagging” in inactive synapses (Okuno et al., 2012). Through extensive *in vitro* and *in vivo* observations, the study shows that this highly regulated protein localizes mainly in inactive synapses by binding an inactive form of CaMKIIβ. Considering these findings, it is possible to create a theoretical framework that explains, at least partially, the occurrence of H-LTD. In summary, a process leading to long-lasting potentiation stimulates the production of PRP mRNA, including Arc mRNA. During its travel along the dendrites, Arc mRNA is first translated in proximity to the plasticity-inducing synapses and then travels to other synapses that did not receive any priming stimulus and are therefore tagged by CaMKIIβ. This mechanism promotes the clearance of surface AMPAR at inactive synapses and helps to maintain the contrast of synaptic weights between these synapses that need to be stimulated and those that need to be weakened. Arc-mediated AMPAR internalization is not the only mechanism responsible for H-LTD.

As stated previously (Brigadski and Leßmann, 2020), the induction of LTP results in the local synthesis and/or release of BDNF. Specifically, a moderate amount of Ca2+ related to E-LTP allows the exocytosis of vesicles containing BDNF. In the case of L-LTP, not only is BDNF released from already docked vesicles, but it is also locally synthesized (Tartaglia et al., 2001). It is worth noting that BDNF is initially synthesized and released as proBDNF, a precursor that is cleaved by the tissue plasminogen activator (tPA)/plasmin protease system to produce the mature protein. Although still a topic of debate, some studies suggest that proBDNF release might mediate LTD in inactive synapses and even synaptic pruning (Woo et al., 2005; Yang et al., 2009). The proBDNF precursor has a high affinity with p75 neurotrophin receptor (p75NTR), which activates a series of pathways that ultimately lead to the reduction of H-Ras activity and to Caspase-3 mediated spine pruning (Guo et al., 2016). Moreover, studies might provide additional evidence for the hypothesis that proBDNF/p75NTR signaling mediates H-LTD, as they have shown that the protein induces the synaptic depression of neighboring, non-coactive spines during spontaneous activity in the hippocampus (Winnubst et al., 2015; Figure 2B).

In summary, the picture that emerges from the literature is that modifying synapses for memory encoding and storage is highly dependent on events that involve a broader area than just a single spine. The molecular pathways described above underlie the capacity of dendrites to associate memory through different forms of cooperative plasticity (Govindarajan et al., 2006; Branco and Häusser, 2010). Due to dendritic compartmentalization, a branch seems to acquire the role of the computational and storage unit of the cell. From a broader perspective, a memory is likely to be stored in a combinatorial fashion, distributed across several dendrites belonging to different neurons, forming what can be considered as the *dendritic engram*.

## 4. Dissecting the role of dendrites in memory engrams with computational models

Computational modeling has been an extremely valuable tool for the investigation of the biophysical and biochemical mechanisms underlying dendritic integration and plasticity, and the functional consequences of these processes on neuronal network activity and behavior. Modeling of dendrites has provided valuable insights into the mechanisms underlying their role in memory encoding and storage. These theoretical investigations suggest that dendritic non-linearities and localized forms of plasticity are enabling neurons and their dendritic domains to learn tasks, and become part of memory engrams. Since dendritic properties drive the synaptic changes which ultimately form the memory engram, these studies highlight the ways in which dendrites can be considered a fundamental aspect of the engram.

### 4.1. Spatial arrangement of synapses affects memory storage

Early computational studies were the first to predict the important role of synapse clustering in memory (Mel, 1992). Multiple studies have since revealed that learning correlates with the emergence of synaptic clusters in neurons which are not explained by chance (Losonczy and Magee, 2006; Takahashi et al., 2012). While these studies provide evidence for the functional co-activation of clustered synapses, the individual synapses participating in those clusters do not always appear to follow an orderly arrangement of the input properties they encode (Jia et al., 2010; Chen et al., 2011; Varga et al., 2011). It has thus not been possible to elucidate the role of synaptic clustering which occurs during memory allocation in the function of memory using neurophysiology experiments alone. Computational studies, on the other hand, have proposed specific memory-related functions and unique advantages conferred by dendritic segregation of signals and synaptic clustering. These studies strongly suggest that dendrites and the arrangement of synapses on them are a key component of the memory engram.

In an early computational study using mathematical models of dendritic integration, (Poirazi and Mel, 2001) found that cells with non-linear subunits have much larger storage capacities compared to traditional connectionist models which summed up synaptic weights at the soma. In particular, the capacity of the neuron to learn distinct patterns was shown to be increased more than 40 times when dendritic non-linearities are taken into account. This capacity was dependent on the spatiotemporal arrangement of the input, namely the clustering of synapses within active dendrites, but not their strength. This study identified the potential of synaptic allocation to shape information storage, which can be modeled as an entire second layer of computation in neurons (Poirazi et al., 2003b). Dendrites can thus expand both the capacity of neural tissue to form engrams, but also their ability to perform more complex computations.

### 4.2. The role of dendritic compartmentalization in memory functions

Computational modeling shows how dendritic dynamics can be utilized by neurons to enable multiple memory-related functions. For example, dendrites may have an important role in enabling “online” learning in neurons. Using a computational model incorporating dendritic spikes, (Wu and Mel, 2009) identified the optimal plasticity rules that maximize online learning and minimize catastrophic forgetting. They found that this can be achieved by a number of plasticity rules which are gated by specific voltage thresholds, target few synapses, and favor binary synaptic weights.

Dendrites may enable storage of distinct features within a neuron (Legenstein and Maass, 2011) used a computational model incorporating branch-strength plasticity (Losonczy et al., 2008) and depolarization-dependent spike-timing dependent plasticity to show that, when dendritic mechanisms are incorporated in the model, dendritic competition arises. Interestingly, this competition leads dendrites to self-organize within the same neuron, and thus each neuron ends up engaged in the storage of separate memory items. Thus, a single neuron can participate in multiple memory engrams and neurons can bind together multiple features of the inputs they receive in their dendrites.

At the neuronal network level, computational modeling shows that the spatial segregation of input streams prevents catastrophic interference when the inputs target separate dendrites (Bono et al., 2017) suggesting that they may prevent the degradation of engrams. The interactions between segregated inputs on dendrites of pyramidal neurons has been studied using a computational model of hippocampal memory (Kaifosh and Losonczy, 2016). The model shows that non-linear dendrites enhance the capacity to store and retrieve similar memories, with CA3 contributing to the decorrelation of engrams, while CA1 pairs engrams with other representations and provides meaningful output during engram recall. The computational modeling studies presented above have provided valuable theories about how neurons maximize their learning potential during memory tasks, and provide testable hypotheses about the role of dendrites in memory, which remain to be studied experimentally.

As mentioned earlier, the late-stage of long-term potentiation and depression depends on structural and biophysical changes in dendrites and neurons, and is protein synthesis-dependent. This has important implications for memory engrams and the interactions between them. In order to study the dendritic aspects of the engram, computational models of plasticity with dendrites are needed. These models take into account dendritic phenomena that span different spatial and temporal scales. These include cooperative plasticity mechanisms (discussed earlier), the plasticity of dendritic excitability via changes in ion channels which alter their coupling to the soma (Losonczy et al., 2008), the synthesis of plasticity related proteins which can be localized in dendrites (Steward and Schuman, 2007) and dendritic homeostatic plasticity, which maintains the stability of network function, regulates synaptic strengths and shapes dendritic responses to input (Turrigiano et al., 1998; Bourne and Harris, 2011).

Using computational modeling of plasticity-protein synthesis (O’Donnell and Sejnowski, 2014) found that spatially patterned protein synthesis allows neurons to selectively encode memories, and to forget other memories, even for simultaneously occurring events that are represented by the same neural ensemble. Synaptic clustering and neural population sparsity were found to be the key factors that enabled this selectivity. In another modeling study of dendritic function, (Bar-Ilan et al., 2013) showed that dendritic inhibition shapes the plasticity of excitatory synapses in dendrites. Inhibition can affect the learning window and properties of STDP, which in turn leads to the fine-tuning of excitatory plasticity in dendrites.

At the neuronal population level, computational modeling showed that dendrites underlie the linking of memories over long time scales (Kastellakis et al., 2016). Memory linking was correlated with synaptic clustering of the respective memories, indicating that dendrites can provide the substrate for engram linking, which is also expressed at the neuronal population level. The authors used a simple protocol involving the encoding of two temporally close memories to show that alterations in excitability levels lead to synaptic clustering of inputs from both memories. Excitability changes thus create a temporal window which enables the mechanistic associations between memory engrams in dendrites.

These studies highlight the potential of dendrites to serve as the sub-cellular substrate of the memory engram and associative memories. An important factor that affects the dendritic allocation of synapses during engram formation is the rate of turnover of synapses in focal points in dendrites. Experiments found that “hotspots” of high synaptic turnover facilitate clustered synaptic spine formation during learning (Frank et al., 2018, p. 20). The authors used computational modeling to show that these hotspots facilitate clustered synaptic spine formation, which in turn increased network sparsity and memory capacity.

Neuromodulation is another important enabler of plasticity. A recent study showed that the neuromodulatory projections from Locus Coeruleus to dorsal CA1 form a key connection for memory linking (Chowdhury et al., 2022) and that blocking this neuromodulatory input to CA1 abolished the linking between two memory engrams, even though the individual neuronal engram sizes remained intact. While the exact effect of neuromodulation on CA1 neurons is not known, the authors used a computational model to show how it is possible for neuromodulation to affect memory engram overlap without changing the size of individual engrams. This is achieved via the combined effect of dendritic plasticity and the plasticity of somatic excitability.

While multiple experimental studies have examined the role of dendrites in memory and behavior, very few studies were able to isolate the effect of dendrites in memory engram formation and to manipulate them. A recent study of memory allocation in the retrosplenial cortex (RSC) found that linking of contextual memory engrams depends on dendrite-specific memory allocation mechanisms (Sehgal et al., 2021). Using novel molecular and optogenetic methods that target recently-activated dendritic segments, the authors found that the linking of two memories activates overlapping dendritic subpopulations and increases synaptic clustering within those dendrites, while unlinked memories do not. Using computational modeling, the authors provided a mechanistic explanation of this observation: learning-induced elevation of dendritic excitability drives population activity overlaps between two engrams and co-clustering of their inputs in common dendrites. The absence of elevated dendritic excitability abolished engram linking during recall, suggesting that dendritic plasticity mechanisms are crucial for the stability of linked engrams. Computational modeling also showed how the reactivation of engram dendrites alone can induce memory linking, suggesting that dendritic mechanisms may be necessary and sufficient for linking temporally close memories. This study therefore lends credence to the idea of a “dendritic memory engram” and further predicts that the degree of dendritic overlap during encoding and recall of two memories can reveal whether two memories are linked.

### 4.3. Dendritic non-linearities in inhibitory neurons affect memory function

While the non-linearities of excitatory neurons have been the focus of a considerable number of experimental and theoretical studies, interneurons are rarely studied for their dendritic properties. Computational modeling has shown, however, that interneuron non-linearities are no less important than pyramidal neuron non-linearities. Using computational modeling, (Tzilivaki et al., 2019) showed that dendrites in Fast Spiking Basket Cells (FSBCs) can exploit the full range of non-linearity in their responses. This allows neurons to take advantage of these non-linearities and greatly expand their computational properties. FSBCs can thus act as 2-stage non-linear integrators, as was previously found to be the case for pyramidal cells. Computational modeling shows that interneuron non-linear dendrites confer major advantages to memory functions such as resource savings by increasing sparsity, improved linking of memories and increased storage capacity.

The consideration that interneurons are computationally no less capable than pyramidal neurons has led to the proposal that interneurons are not merely supporting memory, but are indeed part of the memory engram and have crucial contributions to memory expression (Barron et al., 2017). Inhibitory engrams have been proposed to reduce behavioral response to familiar stimuli as well as prevent inappropriate activation of engrams. Further research in this area is needed to elucidate the role of interneuron dendritic branches in engram formation.

**FIGURE 3:**
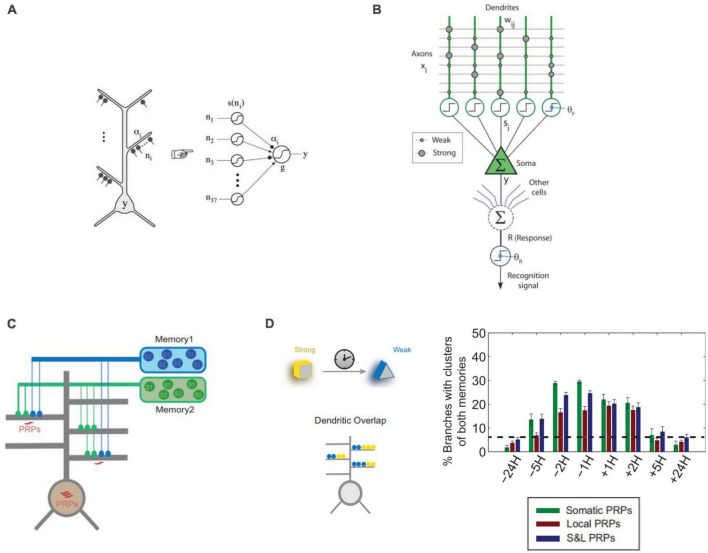
Approaches to computational modeling of dendrites in memory. **(A)** Dendritic branches of CA1 neurons can be modeled as point non-linearities in a 2-layer model of a single neuron (Poirazi et al., 2003b). **(B)** Computational model of the hippocampal Schaffer collateral pathway to CA1 pyramidal neurons incorporating dendritic spikes used to model online learning (Wu and Mel, 2009). **(C)** 2-layer neurons used to model synaptic and neuronal engram formation of 2 temporally separated memories incorporating dendritic plasticity-related protein (PRPs) synthesis and excitability (Kastellakis et al., 2016). **(D)** Results from the model of panel **(C)** show that memories encoded close in time (5H or less) tend to create co-clusters incorporating synapses of both memories in the same dendrite. This holds true whether protein synthesis is only Somatic, only Dendritic (Local) or their combination (S&L). Panels **(A,C,D)** with permissions from authors. Panel **(B)** reprinted from PMC (open access).

In summary, computational modeling has been an important tool for identifying the potential functions of dendrites that are difficult to assess experimentally. Such studies provide valuable insights and theoretical models, and propose experimentally testable hypotheses (Figure 3).

## 5. Outlook: a dendritic view of the engram

The evidence discussed in this article paints a novel view of the neural substrate of memory, whereby the fundamental nature of the engram lies within the dendrites. In this view, dendritic compartmentalization induces synapses to evolve according to the rules of cooperative plasticity and synaptic clustering. This deviates from the classical view of Hebbian synaptic plasticity, in which synapses are considered to have an independent functional role. The consideration of dendritic branches as individual computational and storing units also aligns with the model of cortical associations proposed by Larkum (2012). In this framework, activated branches from the apical and basal trees are proposed to act as a powerful associative mechanism of feed-forward and feedback information at the somatic level.

**FIGURE 4:**
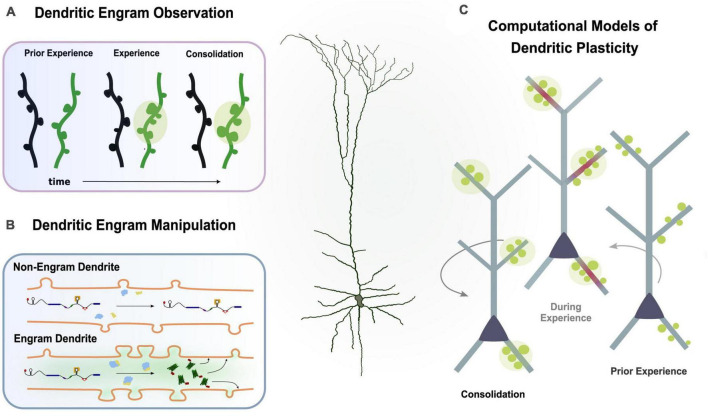
Multiple experimental directions to study the dendritic engram. **(A)** Imaging studies can observe synaptic changes (i.e., spine dynamics, clustering) during learning and memory. **(B)** Protein trafficking allows investigation of plasticity changes in engram dendrites. **(C)** Computational models incorporating findings from experimental studies can simulate learning and memory to infer the functional role of dendrites.

This dendrite-centric view of memory suggests that, by observing and manipulating groups of dendrites, we can influence memory and behavior instead of manipulating populations of neurons. Novel experimental techniques and innovative methods are continuously being developed to allow for the explicit and targeted study of the dendritic engram. In the next paragraphs, we propose some future directions as to how we can further delve into the mysteries of the dendritic engram (Figure 4).

### 5.1. Observing and manipulating dendritic engrams with imaging techniques

Monitoring of synaptic dynamics during learning can provide valuable information about the sub-cellular features of memory engrams. For example, localized synaptic dynamics in dendrites was found to correlate with learning, memory performance and synapse clustering in CCR5 knockout animals (Frank et al., 2018). Thus, probing and monitoring of synaptic dynamics in dendritic domains before and after learning can provide insights to the dendritic contributions to the engram. High-resolution time-lapse imaging coupled with fluorescent biosensors and actuators allows the investigation and manipulation of synaptic structures *in vivo* and *in vitro*. These imaging methods have been used to study the synaptic dynamics of motor learning, visual learning, fear learning or extinction and the priming of future learning (El-Boustani et al., 2018; Ma and Zuo, 2022).

#### 5.1.1. Dendrite-targeting techniques

While typical studies of memory engrams focus on populations of neurons, recent advancements in dendrite-targeting molecular techniques have made it possible to identify and manipulate only the subset of dendrites that are active during memory formation or recall (Sehgal et al., 2021). Using these novel methods, researchers have demonstrated that memory engrams can be defined based on the set of dendrites that are active during the learning or recall of a memory, and that manipulating the excitability of these dendrites can facilitate the linking of memories across time. More studies that selectively target dendrites using such molecular and optogenetic techniques are needed in order to provide new insights into the dendritic properties of the engram.

#### 5.1.2. Tracking plasticity related proteins

Visualizing the trafficking of plasticity-related proteins to and within dendrites using techniques such as fluorescence microscopy, immunohistochemistry, electron microscopy, or molecular methods (Radler et al., 2020) is another way to probe dendritic engrams. Studying the localization of mRNA and/or proteins in dendrites can provide insights into the molecular processes related to plasticity. Future research may explore the manipulation of a gene, either a reporter or a channel protein, by attaching the 3’UTR of a plasticity-related mRNA, such as CaMKIIa or Arc, to this construct (Martin and Ephrussi, 2009). By inducing the expression of this engineered gene in an activity-dependent manner using techniques such as engram labeling (Reijmers et al., 2007), it may be possible to selectively label and/or manipulate the dendrites of neurons undergoing plasticity. This approach could enable the expression of the engineered mRNA only in neurons that have undergone L-LTP. The mRNA would contain the 3’UTR of a PRP mRNA, which would induce its transport and localization only to those dendrites that have been primed by plasticity. This approach could have broad applications, including manipulating dendritic activity during animal behavior to explore their involvement in different tasks.

In addition, mRNA translation can be rapidly influenced by RNA modifications such as RNA methylation (Wang et al., 2015; Flamand and Meyer, 2019). Thus, one future experimental avenue is to interfere with local plasticity mechanisms by manipulating dendritic local RNA modifications which cause rapid changes in mRNA localization, or translation at the dendrites in response to activity (Merkurjev et al., 2018). Future research could explore the feasibility and effectiveness of these approaches, and to refine the design of the engineered mRNA to optimize its activity-dependent expression and localization to dendrites.

Computational studies are increasingly incorporating dendritic function and plasticity into their models. This approach has yielded valuable and testable predictions about the role of dendrites in learning and memory. Despite this progress, there are still gaps in our understanding of the plasticity processes occurring in dendrites. Currently, there is no widely accepted model of plasticity that accurately captures the complex mechanisms underlying these changes, as previously reviewed. This is due in part to the multiple spatial and temporal scales involved in dendritic plasticity, as well as the diverse processes that contribute to it, such as protein synthesis and trafficking, ion channel modifications, signaling cascades, and homeostatic and excitability alterations. Computational modeling holds promise for integrating these diverse phenomena into a cohesive model of dendritic plasticity.

By utilizing computational models, researchers can make predictions about the contribution of dendrites to memory, which can then be experimentally tested to validate the models and gain insights into the underlying mechanisms of dendritic function. Computational models can also generate new hypotheses for *in silico* testing before moving on to experimental testing. This is particularly useful for exploring complex or poorly understood aspects of dendritic function, such as the role of dendritic spikes in information processing or the contributions of various ion channels to dendritic excitability. By combining insights from theoretical and experimental work, the role of dendrites in memory is increasingly being elucidated.

## Data availability statement

The original contributions presented in this study are included in the article/supplementary material, further inquiries can be directed to the corresponding author.

## Author contributions

All authors co-authored this work and approved it for publication.
